# Supplementary material for: Temperature and precipitation affect the water fetching time burden in Sub-Saharan Africa
Source: Nat Commun. 2025 Apr 12;16:3486. doi: 10.1038/s41467-025-58780-9 (PMC11993627; doi:10.1038/s41467-025-58780-9)
Supplement: Supplementary file 1 — Supplementary Information [file 41467_2025_58780_MOESM1_ESM.pdf]

Supplementary Information

Temperature and precipitation affect the water fetching time burden in Sub-Saharan Africa

Abigail Harvey Paulos[1], David A. Carroll II[2], Julie Powers[1], Jake Campolo[3], Daehyun Daniel Kim[1], Avery Cohn[2], Amy J. Pickering[1,4,5*]
Affiliations:
[1] Department of Civil and Environmental Engineering, University of California, Berkeley, United States, 94720
[2] Friedman School of Nutrition Science and Policy, Tufts University, Boston, MA, United States, 02111
[3] Farmer's Business Network, San Carlos, CA, United States
[4] Chan Zuckerberg Biohub, San Francisco, CA, United States
[5] Blum Center for Developing Economies, University of California, Berkeley, Berkeley, CA, United States 94720
*Corresponding author: pickering@berkeley.edu

Table of Contents

Supplementary Methods 1: Fixed effects model specifications **2**

Supplementary Methods 2: Wealth Adjustment **2**

Supplementary Methods 3: Sensitivity Analyses **3**
- Weather Extremes 3

Supplementary Note 1: Household characteristics **3**

Supplementary Note 2: Sensitivity Analyses **7**
- Model Formulation 7
- Spatial First Differences sensitivity analyses 7

Supplementary Note 3: Fixed Effects Models **12**
- Fixed effects models 12
- Comparison between SFD and fixed effects models 12
- Wealth & Electricity Access 13

Supplementary Note 3: Electricity access subgroup analyses **20**

Supplementary References **23**

Supplementary Methods 1: Fixed effects model specifications
Using fixed effects models, we investigated urban status and electricity access as effect modifiers. We included an interaction term in the model to determine the impacts of rural/urban status on the relationship between weather and walk times as supplementary equation (1):

$$WT_i = \beta_0 + \beta_1 WV_{k,i} \times Rural_{urban} + \beta_2 Country_i + \beta_3 County_i + \beta_4 Year_i + \beta_5 Month_i \quad (1)$$

Similarly, we included an interaction term between weather and electricity access status as supplementary equation (2):

$$WT_i = \beta_0 + \beta_1 WV_{k,i} \times Electricity + \beta_2 Country_i + \beta_3 County_i + \beta_4 Year_i + \beta_5 Month_i \quad (2)$$

Supplementary Methods 2: Wealth Adjustment

DHS datasets include a numerical wealth index, which is calculated by DHS from other DHS survey questions such as asset ownership, construction materials used in the home, and access to and type of water and sanitation facilities. The wealth index is calculated for each country-survey individually based on the values of these survey questions, and are not comparable across surveys.

Following the method by Rutstein and Staveteig, we converted the wealth index scores to a comparable scale.[1] We first selected the Kenya 2008-2009 DHS survey to serve as the baseline, as of Sub-Saharan countries included, Kenya had around the median value of the World Bank's Gross National Income per capita at purchasing power parity (GNI/P at PPP). To convert each survey's wealth variable to the same scale, we utilized several anchor points. Four anchor points were selected from asset variables in the DHS surveys: possession of a television, a refrigerator, a car or truck, or a landline telephone. For each of these anchor variables, the wealth score at which 50% of the households possessed that item was selected as an anchor point, as derived from a logistic regression between asset ownership probability and wealth scores. Three more anchor points were derived from the Unsatisfied Basic Needs (UBN) index. For this dataset, UBN values could range from zero to three; a point was added for having no sleeping room or more than three people per sleeping room, having unimproved sanitation facilities, and inadequate floor or wall materials in the dwelling. The percentile of households having 3, 2+, and 1+ points were calculated for each survey, and the wealth values at those percentiles were used as three additional anchor points.

After establishing the anchor points for each survey, we conducted a regression for each non-baseline survey as supplementary equation (3):

$$BA_i = \beta_0 + \beta_1 x SA_i \quad 3$$

where BA are the anchor points for the baseline Kenya 2008-09 survey and SA is the non-baseline survey anchor points. All non-baseline data points were then converted to the baseline wealth scale by multiplying each wealth score by β_1 and adding β_0. We also converted the adjusted wealth scores to a categorical variable representing the quintile of wealth scores to match the DHS-reported wealth score variable.

Because we believe that wealth may modulate the relationship between electricity, weather, and walk times, we conducted fixed effects regressions including wealth quintile as an additional interaction term as supplementary equation (4):

$$WT_i = \beta_0 + \beta_1 WV_{k,i} \times Electricity + Electricity \times Wealth_{quintile} + \beta_2 Country_i + \beta_3 County_i + \beta_4 Year_i + \beta_5 Month_i \quad (4)$$

We also conducted SFD analyses controlling for wealth quintile. Wealth quintile was treated as a categorical variable and values were differenced between adjacent grid cells.

Supplementary Methods 3: Sensitivity Analyses
To evaluate the robustness of our SFD findings, we conducted several sensitivity analyses. GPS coordinates reported in DHS datasets are jittered by up to 10km in rural areas and 2km in urban areas for privacy and anonymity reasons; thus, any data points within 2-10km of our weather grid cell borders may be misclassified and paired with meteorological data of the incorrect grid cell. To analyze the impact of these possible misclassifications on our findings, we removed all data points within 10km or 2 km of grid cell borders in rural and urban areas, respectively, and conducted SFD analyses on this reduced dataset.

While we believe our main SFD analysis is the ideal method for this analysis, as we maintain the maximum amount of variability within each grid cell by selecting pairs between adjacent grid cells and iterating on this approach 1000 times, we also explored the robustness of our findings using other approaches. As a comparison, we calculated the average walk time and weather per grid cell and conducted SFD analyses on this dataset using the following model formulation:
$AWT_i = \beta_0 + \beta_1 * AWV_{k,i}$
where AWT indicates the average walk time per grid cell and AWV indicates the average weather per grid cell of the kth weather variable. We calculated robust standard errors clustered by DHS survey and for each 'channel' of differencing.[2]

Weather Extremes
We explored the impacts of weather extremes on water fetching walk times. We conducted two regressions: one with temperature and precipitation binned by the 75th percentile, and one with both binned at the 90th percentile. Spatial first differences models were used to estimate the effect of recent weather above the 75th and 90th percentiles on water fetching walk times as supplementary equation (5):

$$WT_i = \beta_0 + \beta_1 WE_{k,i} \quad (5)$$

where WE is binary variable that is 1 if recent weather is above the 75th or 90th percentile, and 0 otherwise.

Supplementary Note 1: Household characteristics
Of all data, 67% of GPS points of households were rural and 33% were urban; of rural data, 11% of households had electricity access, 88% did not have electricity, and 1% were missing electricity access information. Overall, 93% of households were in a grid cell with adjacent neighbor(s) to be used in SFD. In rural data 91% had adjacent neighbors, while in urban data 66% had adjacent neighbors; 92% of those with electricity and 85% without had adjacent neighbors. 92% of rural and 85% of urban households had water not in plot/yard, and 55% of rural and 68% of urban had improved sources as defined by the UN.

Overall, walk times vary from 0 to over 900 minutes. The mean walk time across all data is 10 minutes, sd = 36 minutes. We find that mean daily precipitation and temperature increase, and variation in precipitation and temperature decrease, as the length of the lag period increases

(**Supplementary Table 1**). This may be due to surveys being conducted more often in dry seasons.

Supplementary Table 1. Median, 5th and 95th percentiles of mean daily precipitation, maximum temperature, and one-way walk time to water source

	Lag period	n	5th percentile	Median (sd)	95th percentile
Precipitation (cm/week)	7 days	964751	0	1.16 (3.28)	8.59
	30 days	964733	0	1.58 (2.79)	7.53
	90 days	964751	0.01	1.78 (2.25)	6.23
	180 days	964751	0.07	2.16 (1.69)	5.13
	365 days	964751	0.73	2.27 (1.24)	5.00
Temperature (°C)	7 days	972001	21.69	29.19 (4.74)	37.70
	30 days	972001	22.00	29.15 (4.53)	37.27
	90 days	972001	22.08	29.07 (4.31)	36.09
	180 days	967795	22.33	29.36 (4.19)	35.59
	365 days	962577	22.93	29.60 (4.00)	35.77
Walk time (minutes)		846892	0	10 (36)	70

We categorized drinking water sources into improved and unimproved per the Joint Monitoring Program (JMP) definitions. Improved sources include boreholes, protected wells, protected springs, rain water, and piped water; unimproved sources include unprotected wells, unprotected springs, surface water, and vended water. Overall, 47% of households in the data set had access to improved drinking water. A greater proportion of households had access to improved drinking water in urban areas (59%, SE 0.08%) than rural areas (40%, 0.06%) (t-test, $p < 0.001$). Further, improved drinking water access was more common in households with electricity (57% *versus* 42%). These results on access to improved drinking water are consistent with reports from the JMP monitoring reports on Drinking Water Access.[3,4]

Surface water, improved groundwater, and piped water were the most common drinking water sources overall, accounting for 29, 28, and 26% of data respectively. Springs were associated with the greatest walk times, followed by surface water, borewells, shallow wells, vendor water, piped water, and rain water (Supplementary **Fig 1**). We found significant differences between water source usage in rural and urban regions. In urban regions *versus* rural regions, piped water sources were far more common (43% vs 18%), groundwater (borewell or shallow well) was less common (27% vs 39%), and surface water was less common (22% vs 32%). Notably, mean walk times stratified by source type were significantly shorter in urban areas than in rural regions with the exception of protected springs and surface water.

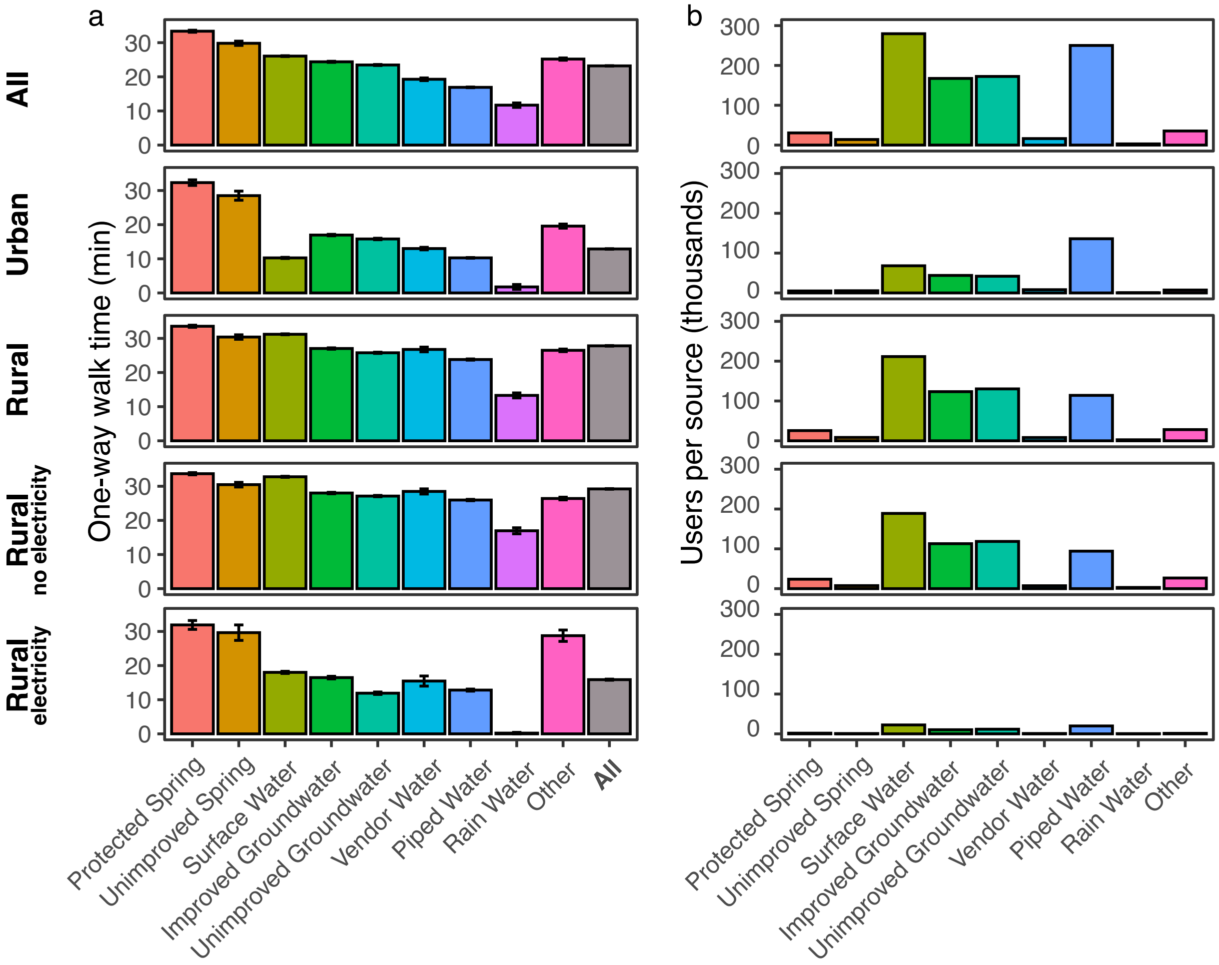

Supplementary Figure 1: Characteristics of households in data set. a) Number of users who reported using each water source type, starting with overall, urban, rural, rural without electricity, and rural households with electricity; b) Mean walk time per water source in the same groups. Errors bars display the standard error of the mean. Improved groundwater includes protected shallow wells and borewells; unimproved ground water includes unprotected shallow wells.

Surveys included by year are shown in Supplementary **Fig 2**. Surveys by region and month are shown in Supplementary **Fig 3**.

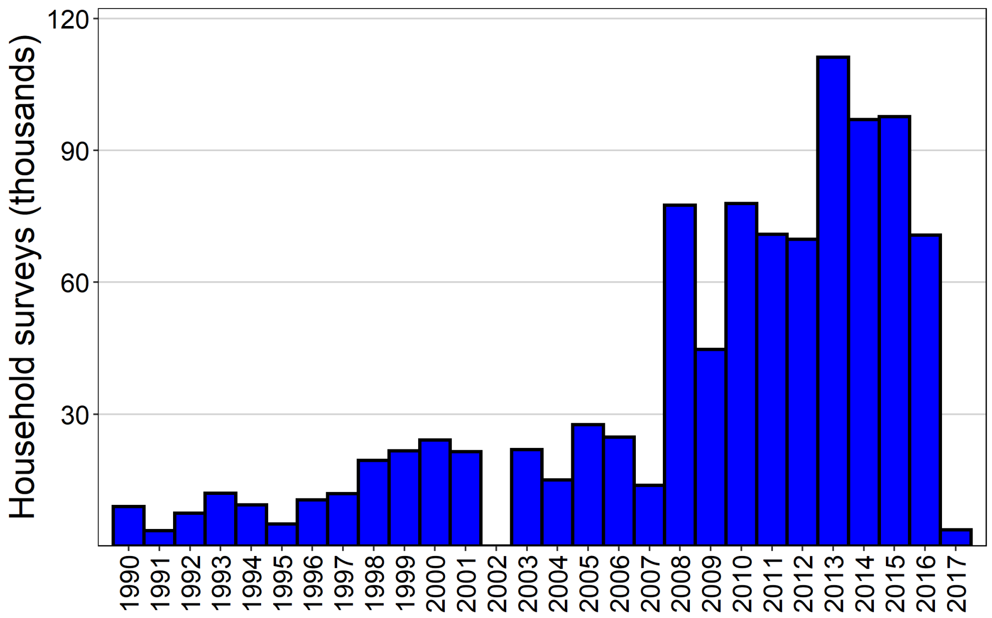

Supplementary Figure 2: Number of household surveys per year.

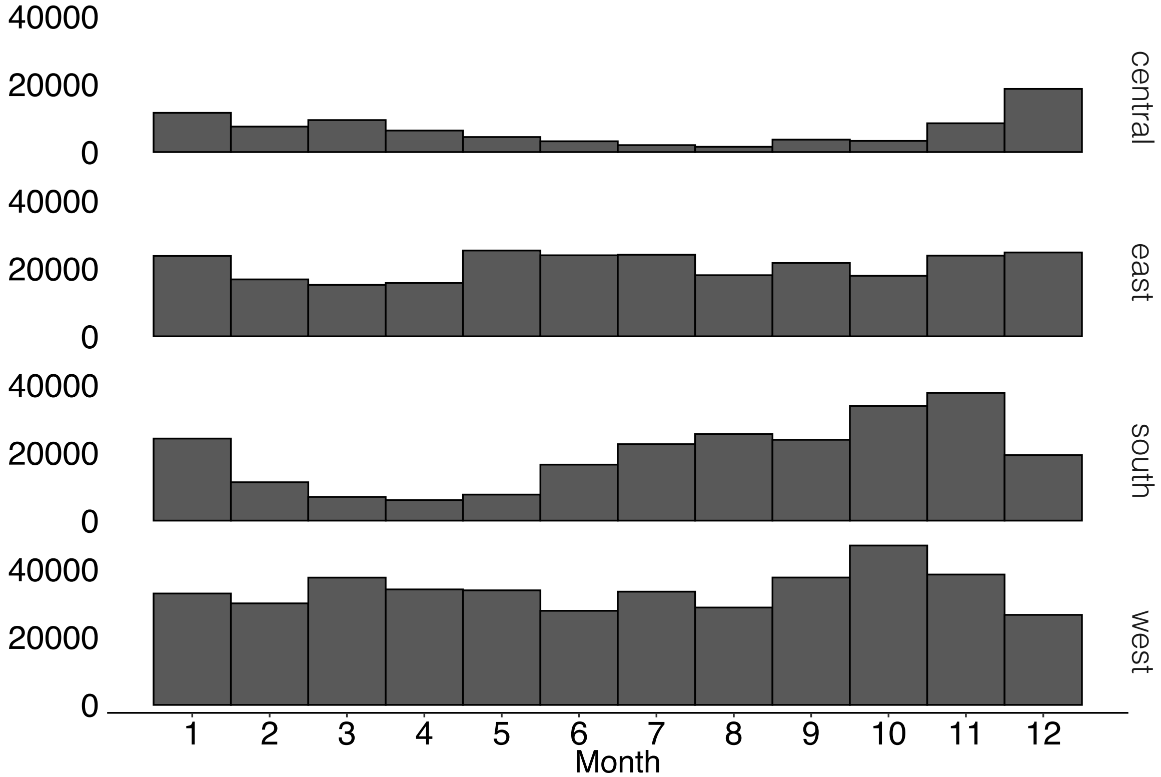

Supplementary Figure 3: Number of household surveys by region and month of the year. Countries are categorized by UN-defined regions in Sub-Saharan Africa.[5]

Supplementary Note 2: Sensitivity Analyses

Model Formulation

We found that a polynomial of order 1 best fit the data in analyses across the majority of time periods and selected this formulation for our main analysis.

Spatial First Differences sensitivity analyses

Supplementary Table 2. Spatial first differences regression results for 365-day lag periods of temperature and precipitation and for west-east and north-south directions. Precipitation results are given in mm/week/min; temperature results are degrees Celsius/min.

		West-east		North-south	
		β (95% CI)	n	β (95% CI)	n
Precipitation	Overall	-3.55 (-3.74, -3.36)	377821	-3.21 (-3.41, -3.01)	368063
	Rural	-5.02 (-5.26, -4.78)	289722	-5.42 (-5.67, -5.17)	281057
	No electricity	-5.89 (-6.15, -5.63)	258190	-5.97 (-6.22, -5.72)	248718
	Electricity	0.32 (-0.27, 0.91)	21517	-0.2 (-0.9, 0.5)	21682
Temperature	Overall	0.76 (0.7, 0.82)	379353	1.2 (1.14, 1.26)	367204
	Rural	0.86 (0.78, 0.94)	293964	1.43 (1.35, 1.51)	282293
	No electricity	0.81 (0.73, 0.89)	261201	1.33 (1.25, 1.41)	250394
	Electricity	0.81 (0.57, 1.05)	22095	0.6 (0.4, 0.8)	21107

Supplementary Table 3. Spatial first differences regression results for 180-day lag periods of temperature and precipitation and for west-east and north-south directions. Precipitation results are given in mm/week/min; temperature results are degrees Celsius/min.

		West-east		North-south	
		β (95% CI)	n	β (95% CI)	n
Precipitation	Overall	-1.63 (-1.71, -1.55)	377821	-1.55 (-1.63, -1.47)	368063
	Rural	-1.82 (-1.92, -1.72)	289722	-1.89 (-1.99, -1.79)	281057
	No electricity	-1.95 (-2.05, -1.85)	258190	-2.07 (-2.18, -1.96)	248718
	Electricity	-0.92 (-1.19, -0.65)	21517	-0.96 (-1.24, -0.68)	21682
Temperature	Overall	0.24 (0.18, 0.3)	380851	0.69 (0.64, 0.74)	368501
	Rural	0.33 (0.26, 0.4)	295285	0.9 (0.84, 0.96)	283369
	No electricity	0.28 (0.21, 0.35)	262461	0.78 (0.71, 0.85)	251411
	Electricity	0.61 (0.41, 0.81)	22145	0.42 (0.25, 0.59)	21150

Supplementary Table 4. Spatial first differences regression results for 90-day lag periods of temperature and precipitation and for west-east and north-south directions. Precipitation results are given in mm/week/min; temperature results are degrees Celsius/min.

		West-east		North-south	
		β (95% CI)	n	β (95% CI)	n
Precipitation	Overall	-0.99 (-1.05, -0.93)	377821	-0.86 (-0.92, -0.8)	368063
	Rural	-1.08 (-1.15, -1.01)	289722	-1.06 (-1.13, -0.99)	281057
	No electricity	-1.14 (-1.21, -1.07)	258190	-1.1 (-1.18, -1.02)	248718
	Electricity	-0.61 (-0.83, -0.39)	21517	-0.76 (-0.98, -0.54)	21682
Temperature	Overall	0.28 (0.23, 0.33)	381951	0.65 (0.61, 0.69)	369591
	Rural	0.32 (0.26, 0.38)	296270	0.84 (0.79, 0.89)	284328
	No electricity	0.28 (0.22, 0.34)	263456	0.76 (0.7, 0.82)	252375
	Electricity	0.55 (0.39, 0.71)	22146	0.46 (0.32, 0.6)	21151

Supplementary Table 5. Spatial first differences regression results for 30-day lag periods of temperature and precipitation and for west-east and north-south directions. Precipitation results are given in mm/week/min; temperature results are degrees Celsius/min.

		West-east		North-south	
		β (95% CI)	n	β (95% CI)	n
Precipitation	Overall	-0.52 (-0.57, -0.47)	377785	-0.45 (-0.49, -0.41)	368063
	Rural	-0.65 (-0.71, -0.59)	289686	-0.57 (-0.62, -0.52)	281057
	No electricity	-0.68 (-0.74, -0.62)	258154	-0.58 (-0.64, -0.52)	248718
	Electricity	-0.32 (-0.49, -0.15)	21517	-0.51 (-0.68, -0.34)	21682
Temperature	Overall	0.56 (0.52, 0.6)	381951	0.72 (0.68, 0.76)	369591
	Rural	0.63 (0.58, 0.68)	296270	0.86 (0.81, 0.91)	284328
	No electricity	0.61 (0.56, 0.66)	263456	0.84 (0.79, 0.89)	252375
	Electricity	0.56 (0.43, 0.69)	22146	0.45 (0.33, 0.57)	21151

Supplementary Table 6. Spatial first differences regression results for 7-day lag periods of temperature and precipitation and for west-east and north-south directions. Precipitation results are given in mm/week/min; temperature results are degrees Celsius/min.

		West-east		North-south	
		β (95% CI)	n	β (95% CI)	n
Precipitation	Overall	-0.18 (-0.21, -0.15)	377821	-0.09 (-0.12, -0.06)	368063
	Rural	-0.23 (-0.26, -0.20)	289722	-0.13 (-0.16, -0.10)	281057
	No electricity	-0.23 (-0.26, -0.20)	258190	-0.14 (-0.18, -0.10)	248718
	Electricity	-0.1 (-0.21, 0.01)	21517	-0.1 (-0.21, 0.01)	21682
Temperature	Overall	0.47 (0.43, 0.51)	381951	0.52 (0.49, 0.55)	369591
	Rural	0.55 (0.51, 0.59)	296270	0.64 (0.6, 0.68)	284328
	No electricity	0.54 (0.5, 0.58)	263456	0.63 (0.59, 0.67)	252375
	Electricity	0.51 (0.38, 0.64)	22146	0.47 (0.35, 0.59)	21151

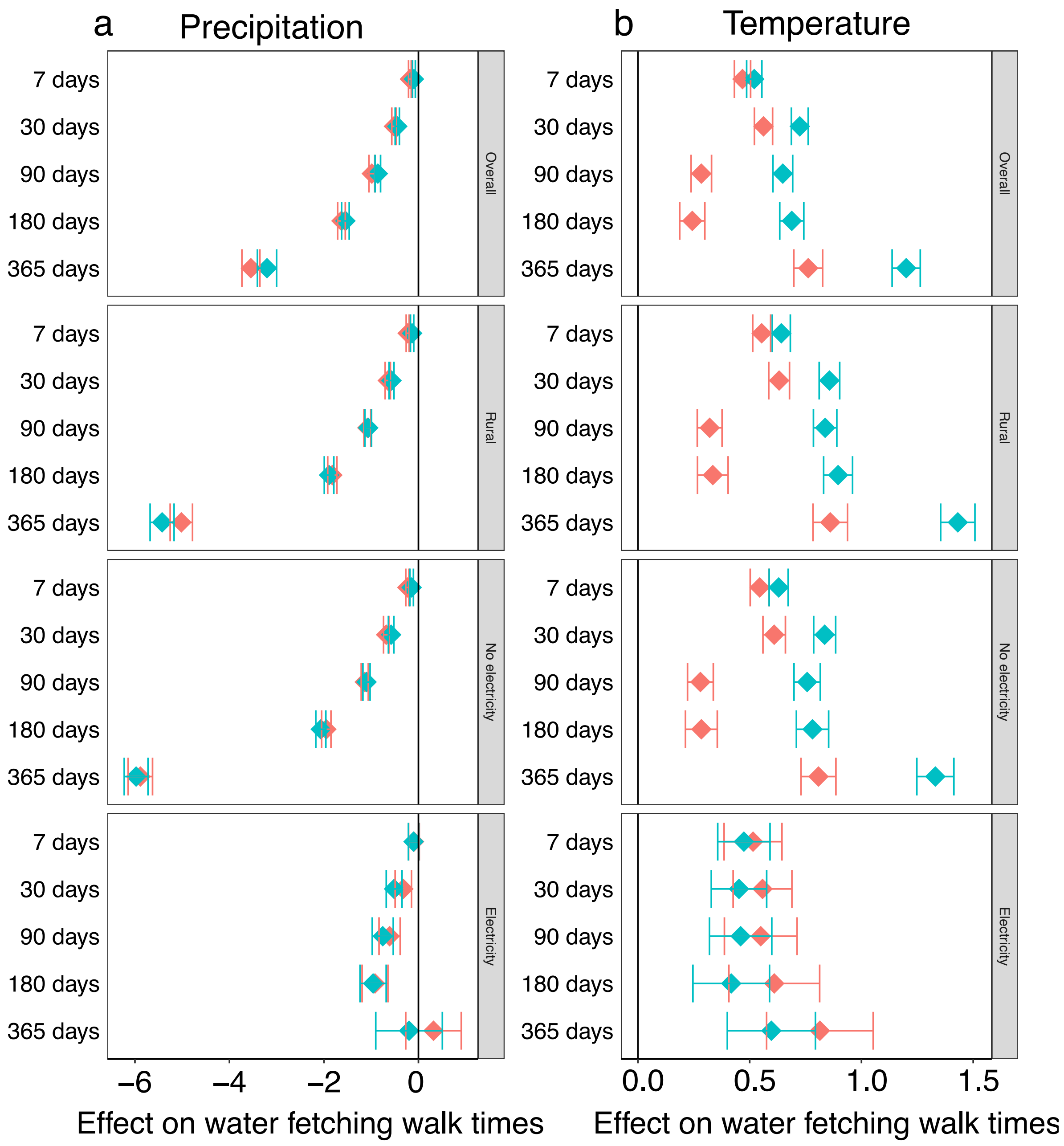

Supplementary Figure 4: Comparison of SFD regression coefficients of the effect of weather on water fetching time by differencing direction, west-east (red circles) *versus* north-south (blue circles). A) Comparison for precipitation. B) Comparison for temperature. Regression coefficients are presented for the 5 time periods. For all models, 95% confidence intervals around the point estimate are presented.

Precipitation above the 75th and 90th percentiles are both associated with decreases in walk time, while temperature above the 75th and 90th percentiles are both associated with increases in walk time (**Supplementary Tables 7 and 8**). The expected directions of effect held for these analyses. However, the magnitudes of the estimates across different directions (west-east and south-north) differ more than our main analysis, suggesting that the magnitudes of effect should be interpreted with caution.

Supplementary Table 7. Spatial first differences results of precipitation and temperature binned by above or below the 75th percentile.

		Est.	p	n	F	Adj. R2
**Precipitation above 75th percentile**	**7 days**	-17.72	0	377823	126.87	0.00
	**30 days**	-26.37	0	377787	206.02	0.00
	**90 days**	-25.15	0	377823	152.55	0.00
	**180 days**	-24.51	0	377823	147.12	0.00
	**365 days**	-20.13	0	377823	52.78	0.00
**Temperature above 75th percentile**	**7 days**	1.79	0	381950	58.95	0.00
	**30 days**	1.80	0	381950	48.35	0.00
	**90 days**	1.99	0	381950	50.67	0.00
	**180 days**	0.88	0	380851	7.82	0.00
	**365 days**	1.27	0	379352	8.70	0.00

Supplementary Table 8. Spatial first differences results of precipitation and temperature binned by above or below the 90th percentile.

		Est.	p	n	F	Adj. R2
**Precipitation above 90th percentile**	**7 days**	-6.35	0	377821	8.99	0.00
	**30 days**	-19.30	0	377785	51.94	0.00
	**90 days**	-17.15	0	377821	32.34	0.00
	**180 days**	-17.52	0	377821	34.17	0.00
	**365 days**	-46.90	0	377821	102.98	0.00
**Temperature above 90th percentile**	**7 days**	0.93	0	381955	9.22	0.00
	**30 days**	1.06	0	381955	11.29	0.00
	**90 days**	0.81	0	381955	6.90	0.00
	**180 days**	0.33	0	380855	1.22	0.00
	**365 days**	0.25	0	379356	0.63	0.00

When we calculate the mean for values within our grid cells rather than selecting pairs between adjacent grid cell, the direction and magnitude of effect are similar to our main analysis results (**Supplementary Table 9**).

Supplementary Table 9. Spatial first differences model results using the mean walk time and weather per grid cell.

		Est	p	n	F	Adj R^2
**Precipitation**	**7 days**	-0.40	0.01	5452	5.44	0.0008
	**30 days**	-0.90	0.00	5450	11.54	0.0019
	**90 days**	-0.90	0.00	5452	6.54	0.0010
	**180 days**	-1.7	0.00	5452	11.29	0.0019
	**365 days**	-5.6	0.00	5452	25.54	0.0045
**Temperature**	**7 days**	0.66	0.00	5530	15.68	0.0026
	**30 days**	0.78	0.00	5530	15.48	0.0026
	**90 days**	0.76	0.00	5530	10.24	0.0017
	**180 days**	0.90	0.00	5502	8.36	0.0013
	**365 days**	1.49	0.00	5459	15.57	0.0027

When we remove data points near grid cell borders, our dataset is reduced by approximately 30%, from nearly 1 million data points to approximately 650,000. Direction and magnitude of effect are similar between these models and our main analysis results (**Supplementary Table 10**).

Supplementary Table 10. Spatial first differences results of precipitation and temperature with households removed that had a GPS coordinate within jittering distance of the border (rural: 5km, urban: 2km).

		Est.	p	n	F	Adj. R2
**Precipitation**	**7 days**	-2.1	0	242438	51.35	0.00
	**30 days**	-5.4	0	242402	132.20	0.00
	**90 days**	-9.7	0	242438	220.38	0.00
	**180 days**	-16.3	0	242438	325.47	0.00
	**365 days**	-30.5	0	242438	190.32	0.00
**Temperature**	**7 days**	0.54	0	245938	276.62	0.00
	**30 days**	0.61	0	245938	237.29	0.00
	**90 days**	0.36	0	245938	62.12	0.00
	**180 days**	0.31	0	245125	32.38	0.00
	**365 days**	0.84	0	244261	172.61	0.00

Supplementary Note 3: Fixed Effects Models

Fixed effects models

We found that across our primary models, elevated precipitation was inversely associated with walk times, while increased temperature was positively associated with walk times (**Supplementary Tables 11-20**). In both model types, the magnitude of effect increased with the length of the lag period, and the largest effects on walk times were found when using the 365-day lag of weather. Between rural and urban households, rural households were more than 4 times as impacted by precipitation and 3 times as impacted by temperature than urban households. Within rural households, households without electricity are more impacted by both precipitation and temperature than those with electricity access. Further, in rural households with electricity access, models predicted a positive association between precipitation and walk times, indicating that an increase in rainfall is related to an increase in walk time for these households, an opposite effect than was observed in all other precipitation models.

Comparison between SFD and fixed effects models

The fixed effects models for precipitation and temperature generally estimated comparable results to the SFD models (**Supplementary Fig 4**). Similar to the SFD models, fixed effects models estimated larger magnitudes of effect for longer time periods. For precipitation overall and in rural areas, the magnitude of effect estimated by fixed effects is larger than estimates using SFD for all time periods. In rural areas with electricity, fixed effects estimate a large effect for the 7, 30, 90, and 180 day time periods, and a similar effect for the 365 day time period compared to SFD estimates. For rural households without electricity, fixed effects results are similar to SFD results for the 7, 30, 90, and 180 day lag periods, while FE and SFD models diverge for the 365-day lag period. Similarly, fixed effects models estimate a larger magnitude of effect than SFD models for temperature in 15 of 20 models. See Tables S9-S18 for fixed effects

regression results. Broadly, the fixed effects models agreed with SFD models on the direction of effect and the increasing magnitude of effect with longer lag periods, while fixed effects estimated larger magnitudes of effects for most models. These regression results support the idea that SFD is better able to control for unobserved confounders and better assess theing and weather.

Wealth & Electricity Access

To explore the impact of wealth on electricity access and susceptibility to short-term weather changes, we conducted fixed effects models including wealth quintile as an interaction term with electricity access. We find a decreased magnitude of effect for precipitation and an increased magnitude of effect for temperature (see **Supplementary Tables 11 – 20**).

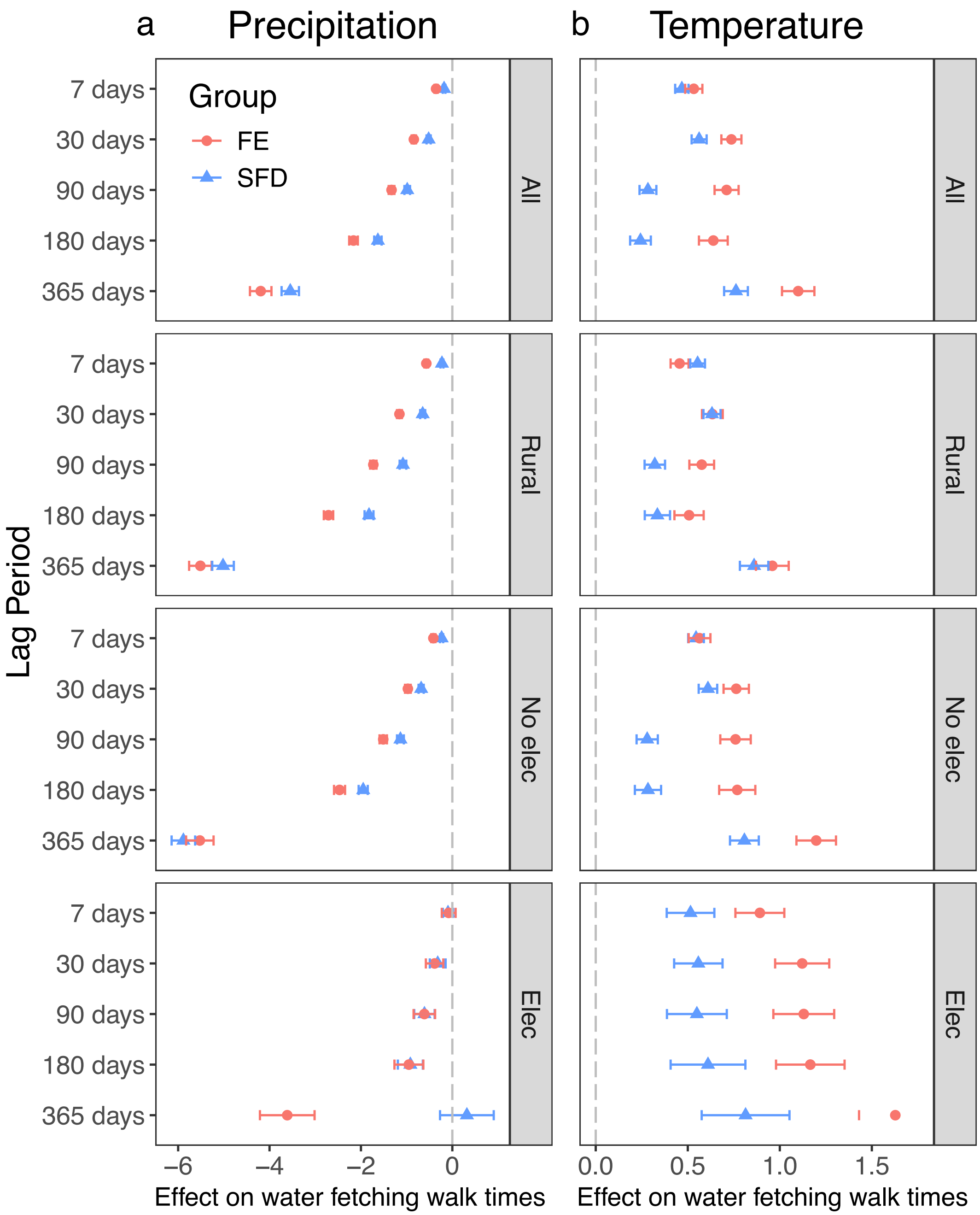

Supplementary Figure 5. Comparison of regression coefficients between spatial first differences (SFD) approach and fixed effects (FE) approach, disaggregated by time period, rural status, and electricity status. A) Comparison for precipitation. B) Comparison for temperature. For all models, 95% confidence intervals around the point estimate are presented.

Supplementary Table 11. Fixed effects regression results of 7-day lag period of precipitation. Precipitation results are given in cm/week/min

	Model 1			Model 2			Model 3			Model 4		
	**Est.**	**95% CI[1]**	**p**	**Est.**	**95% CI**	**p**	**Est.**	**95% CI**	**p**	**Est.**	**95% CI**	**p**
Intercept	12.57	9.49, 15.65	0	45.08	41.26, 48.9	0	53.63	47.65, 59.61	0	37.94	34.86, 41.02	0
Precipitation	-0.4	-0.4, -0.4	0	-0.6	-0.6, -0.6	0	-0.4	-0.4, -0.4	0	-0.04	-0.04, -0.04	0
Rural				Ref.								
Urban				-13.41	-13.63, -13.19	0						
Weather * urban				0.7	0.7, 0.7	0						
No electricity							Ref.			Ref.		
Electricity							-10.08	-10.43, -9.73	0	-5.76	-8.52, -3.00	0
Weather * electricity							0.03	0.03, 0.03	0	0.03	0.03, 0.03	0
Controls	Year, month, country, county			Year, month, country, county			Year, month, country, county			Year, month, country, county, wealth quintile		
n	835536			835534			579863			432203		
F	248.04			289.27			190.99			194.25		
Adj. R^2	0.12			0.13			0.12			0.13		
[1] Indicates 95% confidence intervals												

Supplementary Table 12. Fixed effects regression results of 30-day lag period of precipitation. Precipitation results are given in cm/week/min.

	**Model 1**			**Model 2**			**Model 3**			**Model 4**		
	**Est.**	**95% CI**	**p**	**Est.**	**95% CI**	**p**	**Est.**	**95% CI**	**p**	**Est.**	**95% CI**	**p**
Intercept	14	10.94, 17.06	0	48.88	45.06, 52.7	0	58.55	52.55, 64.55	0	40.2	37.14, 43.26	0
Precipitation	-0.8	-0.8, - 0.8	0	-1.2	-1.2, -1.2	0	-1	-1, -1	0	-0.11	-0.11, - 0.11	0
Rural				Ref.								
Urban				-14.29	-14.53, -14.05	0						
Weather * urban				1.1	1.1, 1.1	0						
No electricity							Ref.			Ref.		
Electricity							-10.7	-11.09, -10.31	0	-6.45	-9.21, - 3.69	0
Weather * electricity							0.06	0.06, 0.06	0	0.06	-0.14, 0.26	0.27
Controls	Year, month, country, county			Year, month, country, county			Year, month, country, county			Year, month, country, county, wealth quintile		
n	835518			835516			579845			432185		
F	250.77			293.04			193.05			196.62		
Adj. R^2	0.12			0.13			0.12			0.13		

Supplementary Table 13. Fixed effects regression results of 90-day lag period of precipitation. Precipitation results are given in cm/week/min.

	Model 1			Model 2			Model 3			Model 4		
	**Est.**	**95% CI**	**p**	**Est.**	**95% CI**	**p**	**Est.**	**95% CI**	**p**	**Est.**	**95% CI**	**p**
Intercept	16.36	13.28, 19.44	0	52.37	48.55, 56.19	0	60.43	54.41, 66.45	0	38.79	35.71, 41.87	0
Precipitation	-1.3	-1.3, -1.3	0	-1.7	-1.7, -1.7	0	-1.5	-1.5, -1.5	0	-0.17	-0.17, -0.17	0
Rural				Ref.								
Urban				-14.49	-14.73, -14.25	0						
Weather * urban				1.3	1.3, 1.3	0						
No electricity							Ref.			Ref.		
Electricity							-11.4	-11.81, -10.99	0	-7.32	-10.08, -4.56	0
Weather * electricity							0.09	-0.11, 0.29	0.18	0.1	-0.1, 0.3	0.16
Controls	Year, month, country, county			Year, month, country, county			Year, month, country, county			Year, month, country, county, wealth quintile		
n	835536			835534			579863			432203		
F	252.95			295			194.54			198.65		
Adj. R^2	0.12			0.13			0.12			0.13		

Supplementary Table 14. Fixed effects regression results of 180-day lag period of precipitation. Precipitation results are given in cm/week/min.

	Model 1			Model 2			Model 3			Model 4		
	**Est.**	**95% CI**	**p**	**Est.**	**95% CI**	**p**	**Est.**	**95% CI**	**p**	**Est.**	**95% CI**	**p**
Intercept	18.76	15.68, 21.84	0	53.99	50.17, 57.81	0	62.71	56.67, 68.75	0	37.88	34.8, 40.96	0
Precipitation	-2.2	-2.2, -2.2	0	-2.7	-2.7, -2.7	0	-2.5	-2.5, -2.5	0	-0.29	-0.49, -0.09	0
Rural				Ref.								
Urban				-15.36	-15.63, -15.09	0						
Weather * urban				1.7	1.7, 1.7	0						
No electricity							Ref.			Ref.		
Electricity							-12.81	-13.28, -12.34	0	-8.34	-11.1, -5.58	0
Weather * electricity							0.15	-0.05, 0.35	0.07	0.16	-0.04, 0.36	0.05
Controls	Year, month, country, county			Year, month, country, county			Year, month, country, county			Year, month, country, county, wealth quintile		
n	835536			835534			579863			432203		
F	254.73			297.06			196.16			200.59		
Adj. R^2	0.12			0.14			0.12			0.14		

Supplementary Table 15. Fixed effects regression results of 365-day lag period of precipitation. Precipitation results are given in cm/week/min.

	Model 1			Model 2			Model 3			Model 4		
	**Est.**	**95% CI**	**p**	**Est.**	**95% CI**	**p**	**Est.**	**95% CI**	**p**	**Est.**	**95% CI**	**p**
Intercept	16.26	13.18, 19.34	0	56.04	52.22, 59.86	0	64.96	58.96, 70.96	0	44.92	41.8, 48.04	0
Precipitation	-4.20	-4.40, -4.00	0	-5.50	-5.70, -5.30	0	-5.50	-5.70, -5.30	0	-0.55	-0.75, -0.35	0
Rural				Ref.								
Urban				-20.8	-21.17, -20.43	0						
Weather * urban				3.90	3.70, 4.10	0						
No electricity							Ref.			Ref.		
Electricity							-13.82	-14.45, -13.19	0	-8.71	-11.51, -5.91	0
Weather * electricity							0.19	-0.01, 0.39	0.03	0.18	-0.02, 0.38	0.04
Controls	Year, month, country, county			Year, month, country, county			Year, month, country, county			Year, month, country, county, wealth quintile		
n	835536			835534			579863			432203		
F	252.8			300.6			196.89			199.19		
Adj. R^2	0.12			0.14			0.12			0.13		

Supplementary Table 16. Fixed effects regression results of 7-day lag period of temperature. Results are given in degrees Celsius/min.

	Model 1			Model 2			Model 3			Model 4		
	**Est.**	**95% CI**	**p**	**Est.**	**95% CI**	**p**	**Est.**	**95% CI**	**p**	**Est.**	**95% CI**	**p**
Intercept	-1.94	-5.13, 1.25	0.12	35.38	30.93, 39.83	0	39.87	33.83, 45.91	0	15.75	12.24, 19.26	0
Temperature	0.53	0.49, 0.57	0	0.46	0.42, 0.5	0	0.56	0.52, 0.6	0	6.7	6.64, 6.76	0
Rural				Ref.								
Urban				-17.29	-18.25, -16.33	0						
Weather * urban				0.2	0.16, 0.24	0						
No electricity							Ref.			Ref.		
Electricity							-18.82	-20.51, -17.13	0	-10.28	-13.4, -7.16	0
Weather * electricity							0.33	0.27, 0.39	0	0.18	0.12, 0.24	0
Controls	Year, month, country, county			Year, month, country, county			Year, month, country, county			Year, month, country, county, wealth quintile		
n	839065			839063			581480			433492		
F	251.78			290.45			192.99			196.07		
Adj. R^2	0.12			0.13			0.12			0.13		

Supplementary Table 17. Fixed effects regression results of 30-day lag period of temperature. Results are given in degrees Celsius/min.

	**Model 1**			**Model 2**			**Model 3**			**Model 4**		
	**Est.**	**95% CI**	**p**	**Est.**	**95% CI**	**p**	**Est.**	**95% CI**	**p**	**Est.**	**95% CI**	**p**
Intercept	-7.35	-10.6, -4.1	0	31.36	26.89, 35.83	0	36.27	30.21, 42.33	0	8.03	4.42, 11.64	0
Temperature	0.74	0.7, 0.78	0	0.63	0.59, 0.67	0	0.76	0.7, 0.82	0	9.5	9.44, 9.56	0
Rural				Ref.								
Urban				-18.78	-19.78, -17.78	0						
Weather * urban				0.26	0.22, 0.3	0						
No electricity							Ref.			Ref.		
Electricity							-19.64	-21.42, -17.86	0	-10.91	-14.09, -7.73	0
Weather * electricity							0.36	0.3, 0.42	0	0.2	0.14, 0.26	0
Controls	Year, month, country, county			Year, month, country, county			Year, month, country, county			Year, month, country, county, wealth quintile		
n	839065			839063			581480			433492		
F	252.93			291.61			193.75			197		
Adj. R^2	0.12			0.13			0.12			0.13		

Supplementary Table 18. Fixed effects regression results of 90-day lag period of temperature. Results are given in degrees Celsius/min.

	**Model 1**			**Model 2**			**Model 3**			**Model 4**		
	**Est.**	**95% CI**	**p**	**Est.**	**95% CI**	**p**	**Est.**	**95% CI**	**p**	**Est.**	**95% CI**	**p**
Intercept	-6.71	-10.02, -3.4	0	32.1	27.57, 36.63	0	36.01	29.91, 42.11	0	1.93	7.52	3.74, 11.3
Temperature	0.71	0.65, 0.77	0	0.58	0.52, 0.64	0	0.76	0.7, 0.82	0	0.04	9.9	9.82, 9.98
Rural				Ref.								
Urban				-21.81	-22.83, -20.79	0						
Weather * urban				0.36	0.32, 0.4	0						
No electricity							Ref.			Ref.		
Electricity							-20	-21.9, -18.1	0	1.64	-10.57	-13.78, -7.36
Weather * electricity							0.37	0.31, 0.43	0	0.03	0.2	0.14, 0.26
Controls	Year, month, country, county			Year, month, country, county			Year, month, country, county			Year, month, country, county, wealth quintile		
n	839065			839063			581480			433492		
F	251.67			290.74			192.89			196.35		
Adj. R^2	0.12			0.13			0.12			0.13		

Supplementary Table 19. Fixed effects regression results of 180-day lag period of temperature. Results are given in degrees Celsius/min.

	Model 1			Model 2			Model 3			Model 4		
	**Est.**	**95% CI**	**p**	**Est.**	**95% CI**	**p**	**Est.**	**95% CI**	**p**	**Est.**	**95% CI**	**p**
Intercept	-6.12	-9.61, -2.63	0	32.69	28.06, 37.32	0	35.19	29.02, 41.36	0	1.97	13.23	9.37, 17.09
Temperature	0.64	0.58, 0.7	0	0.51	0.45, 0.57	0	0.77	0.69, 0.85	0	0.04	8.40	8.32, 8.48
Rural				Ref.								
Urban				-22.88	-23.9, -21.86	0						
Weather * urban				0.4	0.36, 0.44	0						
No electricity							Ref.			Ref.		
Electricity							-20.81	-22.83, -18.79	0	1.68	-10.97	-14.26, -7.68
Weather * electricity							0.4	0.34, 0.46	0	0.04	0.21	0.13, 0.29
Controls	Year, month, country, county			Year, month, country, county			Year, month, country, county			Year, month, country, county, wealth quintile		
n	836039			836037			579529			433492		
F	250.57			289.87			192.31			195.18		
Adj. R^2	0.12			0.13			0.12			0.13		

Supplementary Table 20. Fixed effects regression results of 365-day lag period of temperature. Results are given in degrees Celsius/min.

	Model 1			Model 2			Model 3			Model 4		
	**Est.**	**95% CI**	**p**	**Est.**	**95% CI**	**p**	**Est.**	**95% CI**	**p**	**Est.**	**95% CI**	**p**
Intercept	-19.23	-22.86, -15.6	0	19.66	14.94, 24.38	0	22.99	16.7, 29.28	0	1.65	-2.39, 5.69	0.21
Temperature	1.1	1.04, 1.16	0	0.96	0.9, 1.02	0	1.2	1.12, 1.28	0	12.3	12.2, 12.4	0
Rural				Ref.								
Urban				-21.84	-22.92, -20.76	0						
Weather * urban				0.36	0.32, 0.4	0						
No electricity							Ref.					
Electricity							-21.75	-23.81, -19.69	0	-11.73	-15.04, -8.42	0
Weather * electricity							0.43	0.37, 0.49	0	0.23	0.15, 0.31	0
Controls	Year, month, country, county			Year, month, country, county			Year, month, country, county			Year, month, country, county, wealth quintile		
n	831992			831990			576339			432789		
F	251.85			290.66			192.95			195.71		
Adj. R^2	0.12			0.13			0.12			0.13		

Supplementary Note 3: Electricity access subgroup analyses

Community Electricity Access

We hypothesized that electricity access in the community, even without access in the home, may be able to alleviate the impacts of climate on water fetching for all community members. To examine this question, we stratified the data by community electricity access and conducted SFD analyses (in addition to the household level stratification also presented). We defined community electricity access as 'available' if any households in the DHS-reported cluster reported electricity access in their household, and as 'unavailable' if no households in the cluster reported electricity access. A limitation of this approach is that not all households within a cluster were surveyed, meaning we may miss households with an electrical connection and thus underestimate the prevalence of community electricity access. Overall, 39% of rural households in the data had community electricity access. We found a 1cm increase in weekly precipitation over the last 365 days resulted in a 3.7 minute (95% CI: -8.1, 0.07 cm/week/min) decrease in walk time for those with community electricity access and a 7.2 minute (-11.6, -0.27) decrease for those without, although these findings are not statistically significant; we find no differences by temperature or CDD. The median walk time was greater in households without community electricity access (20 min *versus* 10 min) (**Supplementary Table 21**).

Households with community electricity access still had significantly higher wealth than those without ($p < 0.001$); however, the mean difference in wealth score between these groups was much less than when considering household electricity access. Approximately 50% of households with community electricity access had a wealth score below the SSA median, and 35% below the rural SSA median.

Supplementary Table 21. Spatial first differences regression results for 365-day lag by community electricity status. Precipitation results are given in cm/week/min; temperature results are degrees Celsius/min.

		West-east		North-south	
		β (95% CI)	n	β (95% CI)	n
Precipitation	Electricity	-3.0 (-3.1, -2.9)	85964	-0.37 (-3.8, -3.6)	83303
	No electricity	-6.3 (-6.4, -6.2)	169218	-6.3 (-6.3, -6.1)	161520
Temperature	Electricity	0.78 (0.74, 0.82)	86906	1.26 (1.22, 1.29)	83437
	No electricity	0.85 (0.82, 0.89)	171073	1.21 (1.18, 1.24)	163145

Supplementary Table 22. Mean weather by climate zone and time period.

		Tropical		Temperate		Arid	
		mean	sd	mean	sd	mean	sd
Precipitation	7 days	30	40	21	28	7.5	16
	30 days	130	130	88	93	34	60
	90 days	350	310	240	240	110	160
	180 days	680	440	450	370	250	240
	365 days	1400	640	1000	300	530	220
Temperature	7 days	29	3.8	24	3.4	33	4.9
	30 days	29	3.6	24	3.1	33	4.6
	90 days	29	3.3	24	3.0	33	4.2
	180 days	30	3.0	24	3.0	33	3.9
	365 days	30	2.9	24	2.2	33	3.7

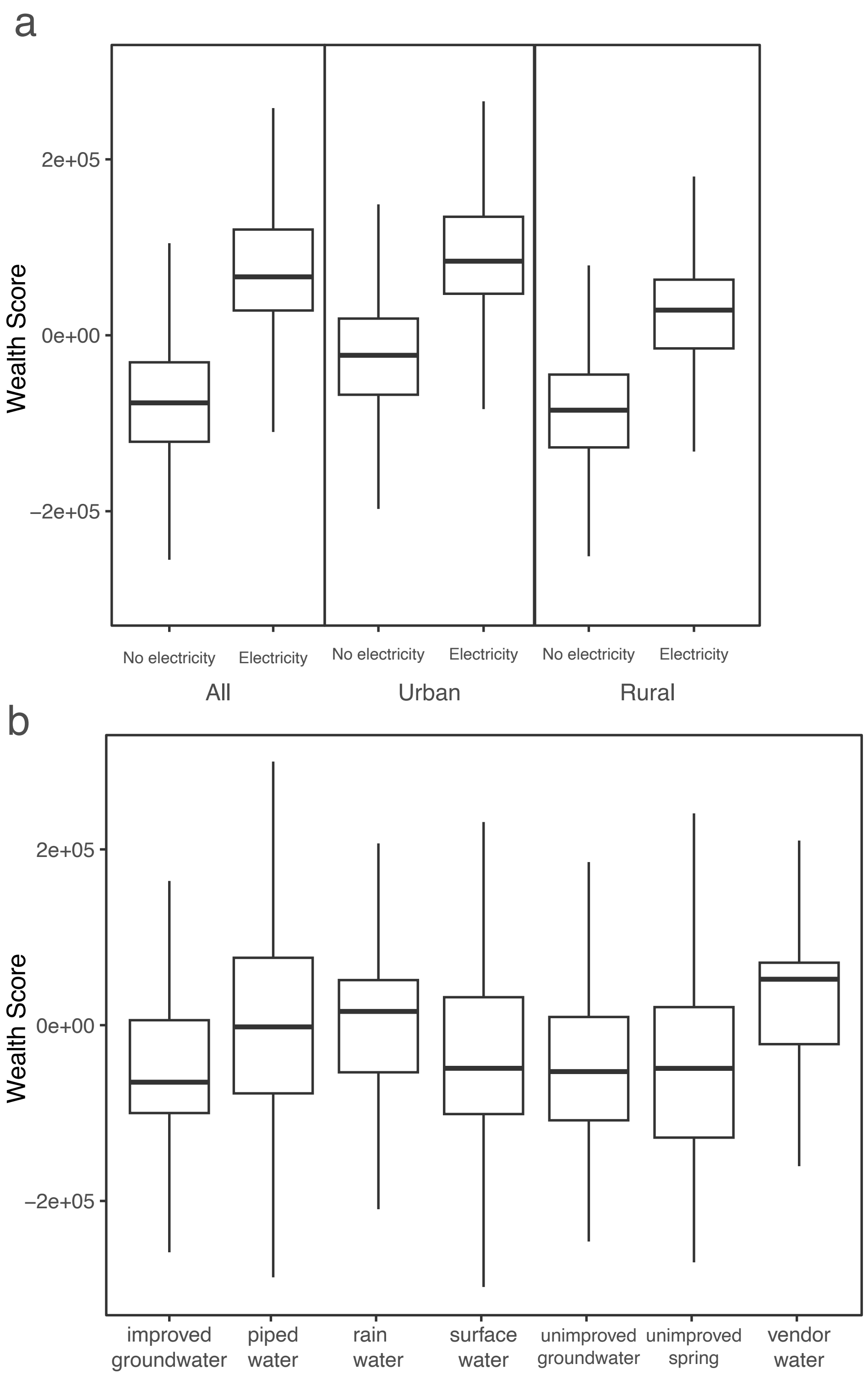

Supplementary Figure 6: Wealth scores by urbanicity and water source. A) Wealth scores by urbanicity. B) Wealth scores by water source. The horizontal lines represent the median, box bounds represent the 25th – 75th percentile, and whiskers represent the 5th – 95th percentile.

Supplementary References

1. Shea O. Rutstein & Sarah Staveteig. Making the Demographic and Health Surveys Wealth Index Comparable. (2014).
2. Druckenmiller, H. & Hsiang, S. *Accounting for Unobservable Heterogeneity in Cross Section Using Spatial First Differences*. (2019).
3. *Progress on Drinking Water, Sanitation and Hygiene: 2017 Update and SDG Baselines. (2017).* World Health Organization (WHO) and the United Nations Children's Fund (UNICEF)
4. *Progress On Household Drinking Water, Sanitation and Hygiene: 2000-2020: Five Years into the SDGs*. (2021). World Health Organization (WHO) and the United Nations Children's Fund (UNICEF)
5. UNSD — Methodology. https://unstats.un.org/unsd/methodology/m49/#geo-regions.
