# Supplementary material for: Temperature and precipitation affect the water fetching time burden in Sub-Saharan Africa
Source: Nat Commun. 2025 Apr 12;16:3486. doi: 10.1038/s41467-025-58780-9 (PMC11993627; doi:10.1038/s41467-025-58780-9)
Supplement: Supplementary file 2 — Reporting Summary [file 41467_2025_58780_MOESM2_ESM.pdf]

nature portfolio

Corresponding author(s): Amy Pickering

Last updated by author(s): 09-26-2024

Reporting Summary

Nature Portfolio wishes to improve the reproducibility of the work that we publish. This form provides structure for consistency and transparency in reporting. For further information on Nature Portfolio policies, see our Editorial Policies and the Editorial Policy Checklist.

Statistics

For all statistical analyses, confirm that the following items are present in the figure legend, table legend, main text, or Methods section.

n/a	Confirmed	
☒	☐	The exact sample size (n) for each experimental group/condition, given as a discrete number and unit of measurement
☒	☐	A statement on whether measurements were taken from distinct samples or whether the same sample was measured repeatedly
☒	☐	The statistical test(s) used AND whether they are one- or two-sided<br>*Only common tests should be described solely by name; describe more complex techniques in the Methods section.*
☒	☐	A description of all covariates tested
☒	☐	A description of any assumptions or corrections, such as tests of normality and adjustment for multiple comparisons
☒	☐	A full description of the statistical parameters including central tendency (e.g. means) or other basic estimates (e.g. regression coefficient) AND variation (e.g. standard deviation) or associated estimates of uncertainty (e.g. confidence intervals)
☒	☐	For null hypothesis testing, the test statistic (e.g. F, t, r) with confidence intervals, effect sizes, degrees of freedom and P value noted<br>*Give P values as exact values whenever suitable.*
☒	☐	For Bayesian analysis, information on the choice of priors and Markov chain Monte Carlo settings
☒	☐	For hierarchical and complex designs, identification of the appropriate level for tests and full reporting of outcomes
☒	☐	Estimates of effect sizes (e.g. Cohen's d, Pearson's r), indicating how they were calculated

Our web collection on statistics for biologists contains articles on many of the points above.

Software and code

Policy information about availability of computer code

Data collection	*Provide a description of all commercial, open source and custom code used to collect the data in this study, specifying the version used OR state that no software was used.*
Data analysis	*Provide a description of all commercial, open source and custom code used to analyse the data in this study, specifying the version used OR state that no software was used.*

For manuscripts utilizing custom algorithms or software that are central to the research but not yet described in published literature, software must be made available to editors and reviewers. We strongly encourage code deposition in a community repository (e.g. GitHub). See the Nature Portfolio guidelines for submitting code & software for further information.

Data

Policy information about availability of data

All manuscripts must include a data availability statement. This statement should provide the following information, where applicable:

- Accession codes, unique identifiers, or web links for publicly available datasets
- A description of any restrictions on data availability
- For clinical datasets or third party data, please ensure that the statement adheres to our policy

All data are available at https://github.com/abharv52/Water_fetching_climate

Research involving human participants, their data, or biological material

Policy information about studies with human participants or human data. See also policy information about sex, gender (identity/presentation), and sexual orientation and race, ethnicity and racism.

Reporting on sex and gender	We report that females were responsible for water collection in 77% of households in this study.
Reporting on race, ethnicity, or other socially relevant groupings	n/a
Population characteristics	Demographic and Health Surveys are conducted with the head of household.
Recruitment	n/a
Ethics oversight	n/a All analyses used publicly available data.

Note that full information on the approval of the study protocol must also be provided in the manuscript.

Field-specific reporting

Please select the one below that is the best fit for your research. If you are not sure, read the appropriate sections before making your selection.

☐ Life sciences ☒ Behavioural & social sciences ☐ Ecological, evolutionary & environmental sciences

For a reference copy of the document with all sections, see nature.com/documents/nr-reporting-summary-flat.pdf

Behavioural & social sciences study design

All studies must disclose on these points even when the disclosure is negative.

Study description	Analysis of publicly available, de-identified household survey data to assess relationship between weather and water fetching time, using a spatial first differences modeling approach. The approach is quantitative.
Research sample	The sample consists of households enrolled into nationally representative survey cohorts by the Demographic and Health Survey program across countries in SSA. Survey data with available GPS coordinates for household clusters were included in the study. This data source was selected because it provides data across a large number of countries in SSA, including information on water fetching walk time, electricity access, and other covariates of interest.
Sampling strategy	n/a; pre-existing dataset used for the analysis
Data collection	n/a; pre-existing dataset used for the analysis
Timing	Surveys are from the time period of 1990-2017
Data exclusions	Data points with missing walk time to water source were excluded from the dataset. Data points with GPS coordinates falling outside their respective country by greater than 10km were also excluded; country extents were determined from the UIA World Country Boundaries. We also excluded data with a survey date that fell outside the survey data collection dates listed by DHS for a particular survey round, with a two-month buffer around start and end dates.
Non-participation	n/a
Randomization	n/a

Reporting for specific materials, systems and methods

We require information from authors about some types of materials, experimental systems and methods used in many studies. Here, indicate whether each material, system or method listed is relevant to your study. If you are not sure if a list item applies to your research, read the appropriate section before selecting a response.

Materials & experimental systems

n/a	Involved in the study
☒	☐ Antibodies
☒	☐ Eukaryotic cell lines
☒	☐ Palaeontology and archaeology
☒	☐ Animals and other organisms
☒	☐ Clinical data
☒	☐ Dual use research of concern
☒	☐ Plants

Methods

n/a	Involved in the study
☒	☐ ChIP-seq
☒	☐ Flow cytometry
☒	☐ MRI-based neuroimaging

Plants

Seed stocks	n/a
Novel plant genotypes	n/a
Authentication	n/a
